# Human placental extract ameliorates methotrexate-induced hepatotoxicity in rats via regulating antioxidative and anti-inflammatory responses

**DOI:** 10.1007/s00280-021-04349-4

**Published:** 2021-09-10

**Authors:** Mamdooh Ghoneum, Mohamed S. A. El-Gerbed

**Affiliations:** 1 Department of Surgery, Charles R. Drew University of Medicine and Science, 1621 E. 120th Street, Los Angeles, CA 90059 USA; 2 Department of Surgery, University of California Los Angeles, Los Angeles, CA 90095 USA; 3 Department of Zoology, Faculty of Science, Damanhour University, Damanhour, Egypt

**Keywords:** Human placental extract, Methotrexate, Liver toxicity, Histopathology, Lipid peroxidation, Cytokines

## Abstract

**Purpose:**

Methotrexate (MTX) induces hepatotoxicity, limiting its clinical efficacy as a widely known chemotherapy drug. In the current study, we examined the protective effect of human placenta extract (HPE) against MTX-induced liver damage in rats, as well as its ability to regulate antioxidative and anti-inflammatory liver responses.

**Methods:**

Male rats were orally administered MTX at a daily dose of 5 mg/kg-body-weight in the presence or absence of HPE (10.08 mg/kg) for 2 weeks. We measured the biological effects of MTX and HPE on the levels of liver enzymes, lipid profile, lipid peroxidation, oxidative stress biomarkers, and cytokines [tumor necrosis factor alpha (TNF-α), interleukin-6 (IL-6), and interleukin-10 (IL-10)]. In addition, histological examination and histopathological scoring of liver tissues were performed.

**Results:**

MTX-treated rats showed significantly increased (*p* < 0.001) liver enzyme levels for aspartate aminotransferase (AST), alanine aminotransferase (ALT), alkaline phosphatase (ALP), total bilirubin, total cholesterol, and triglyceride levels. However, HPE supplementation in MTX-treated rats significantly decreased (*p* < 0.001) these elevated levels. HPE supplementation also significantly reduced the oxidative stress biomarker malondialdehyde (MDA), reversed the reduction in glutathione (GSH), and markedly increased the antioxidant enzyme activities of catalase (CAT) and superoxide dismutase (SOD) in the livers of MTX-treated rats. Furthermore, HPE supplementation significantly decreased the MTX-elevated levels of the pro-inflammatory cytokines TNF-α, IL-6, and IL-10. Histopathological examinations showed that MTX produced severe cellular damage and inflammatory lesions in liver tissues, while treatment with HPE improved hepatic histologic architecture.

**Conclusion:**

HPE has the ability to ameliorate methotrexate-induced liver injury in rats by mechanisms that include boosting antioxidative responses and down-regulating MDA and pro-inflammatory cytokine production.

## Introduction

Methotrexate (MTX), a dihydrofolate reductase inhibitor, is an anti-metabolite used as a chemotherapeutic agent against different types of malignancies [1–3]. MTX is also used to treat a variety of other diseases, including autoimmune diseases, such as rheumatoid arthritis, psoriasis, inflammatory bowel disease, and vasculitis [4–7]. However, MTX induces neurotoxicity [8–11], nephrotoxicity [12], and hepatotoxicity [13, 14], with MTX being linked to a raised hazard of liver damage, cirrhosis, and fibrosis. MTX acts as a dihydrofolic acid analog that binds to the dihydrofolic acid reductase enzyme by inhibiting the synthesis of tetrahydrofolate, which is required for DNA synthesis. Administration of MTX results in an excess output of reactive oxygen species (ROS), which excite many of the pathological processes [15, 16].

Many risk factors can increase the hepatotoxic effect of MTX, including age, duration of exposure to MTX and its cumulative dose, alcohol consumption, history of non-alcoholic steatohepatitis, diabetes, obesity, hepatitis B or C virus infection, and hepatotoxic drugs [17, 18]. Furthermore, non-alcoholic fatty liver disease, in which hepatic steatosis is observed without heavy alcohol consumption [19], has also been linked to MTX therapy [20, 21]. As a serious side effect even at low doses, MTX can lead to hepatic fibrosis and cirrhosis. Low-dose MTX use in psoriasis was found to have a 7% risk of cirrhosis development, and transaminase elevations up to three times normal were seen in 8% of the patients monitored [22]. In addition, Sakthiswary et al. reported that the cumulative dose of MTX was found to have a significant positive correlation with the alanine transaminase (ALT) level [23].

Recent studies have reported the effectiveness of several agents against MTX-induced liver damage, including casticin, myricetin [24], and berberine [11], a natural isoquinoline alkaloid that can be isolated from Coptis chinensis. In the current study, we examined the protective effect of human placenta extract (HPE) against MTX-induced hepatotoxicity in rats. The placenta is a unique organ, connecting the wall of a uterus to a growing fetus. The placenta allows the developing fetus to intake nutrients, eliminate waste, exchange gases, and thermo-regulate through the mother’s blood. This organ interchanges substances between the maternal and fetal blood streams without mixing the two streams or allowing corporeal contact between them [25]. It has become evident that human placenta is the source of a large number of biologically active molecules [26], such as hepatocyte growth factor (HGF) [27], epidermal growth factor (EGF) [28], and transforming growth factor-a (TGF-a) [29]. HPE also contains l-tryptophan as a main antioxidant constituent [30]. In addition, HPE has been used to provide multiple beneficial therapeutic effects, including wound healing [31], health status improvement in elderly subjects [32], and stress reduction [33]. HPE was also reported to improve hepatic damage via liver regeneration [34] and to decrease inflammatory response and apoptosis [35]. These results motivated the present study which aimed to evaluate the protective effects of HPE on MTX-induced hepatotoxicity and to investigate its regulatory antioxidative and anti-inflammatory effects on the liver of rats.

## Materials and methods

### Animals

Forty male albino rats of the Wistar strain (*Rattus norvegicus*) were used in this study. Rats 3 months old (weighing 150 ± 4 g) were purchased from the Laboratory Animal Breeding Colony of the National Research Centre, Dokki Giza, Egypt. Rats were allowed to acclimate for 1 week before the study’s start. Rats were housed in plastic cages under natural room temperature (26 ± 2 °C) with a relative humidity of 50 ± 5% and 12 h dark/light cycle. Food and water were available ad libitum. Food consisted of pellets that were 54% carbohydrate, 3% fat, 26% protein, and 17% vitamins and minerals.

### Drugs and chemicals

MTX ampoules were administered as the clinical formulation. MTX was manufactured by the Hikma Specialized Pharmaceutical Company, Cairo, Egypt. HPE is commercially known as Laennec. Laennec was obtained from YHB Pharma Company, Cairo, Egypt. Each ampoule is water-soluble and consists of a 2 ml solution containing 112 mg of human placenta. All other chemicals used were analytical grade.

### Study design

Rats were randomly divided into four groups with ten animals per group as follows: Group 1 (control): untreated; Group 2 (MTX): injected intraperitoneally (i.p.) with MTX at a dose level of 5 mg/kg-body-weight in physiological saline for 5 days according to [36]; Group 3 (HPE): injected subcutaneously with HPE at a dosage of 10.08 mg/kg-body-weight (equivalent to the therapeutic dose for humans) for 2 weeks [37]; Group 4 (MTX plus HPE): treated with MTX at the same dose and duration as Group 2 and simultaneously treated with HPE at the same dose and duration as Group 3.

At the end of the experimental period, all rats were anesthetized by intravenously administering an overdose of 30–50 mg/kg pentobarbital and then sacrificed by cervical dislocation. Liver tissues were quickly removed and washed using an ice-cold saline solution, then they were cut into small pieces. The first part for homogenates was homogenized in potassium phosphate buffered (pH 7.4), then centrifuged to obtain the supernatants which were kept at -80^0^C until analyzed. The second part of liver tissues were excised and fixed in 10% neutral formalin solution for histopathological analysis.

### Biochemical analyses

#### Liver function

The activities of both AST and ALT and were measured using colorimetric assay of Gella et al. [38]. Alkaline phosphatase (ALP) was assayed according to the method of Kind and King [39]. Serum total bilirubin was determined according to Kaplan et al. [40]. Total cholesterol (TC) and triglyceride (TG) levels were analyzed using diagnostic kits according to Burtis et al. [41].

#### Liver lipid peroxidation

Liver tissue homogenates were analyzed using the spectrophotometric method according to the manufacturer’s protocol of Biodiagnostic, Egypt. The malondialdehyde (MDA) content was assayed in the form of thiobarbituric acid-reactive substances (TBARS) in liver according to the method described previously in [42]. Briefly, 500 µl of sample was added to 1 ml of trichloroacetic acid (TCA 15%) and centrifuged at 3000 rpm for 10 min. 1 ml of supernatant was mixed with 500 µl of thiobarbituric acid (TBA 0.7%), heated in boiling water bath for 10 min, and cooled, and the color was read at 532 nm. The TBARS level was calculated against control according to the following equation: TBARS level (nmol/ml) = Absorbance/0.156. The majority of TBARS are MDA, and thus the concentration of MDA in the sample homogenate was expressed as nmol MDA/mg protein. The results were calculated using an index of absorption for MDA using a molar extinction coefficient 1.56 × 10^5^/M/cm.

#### Endogenous antioxidant activities

Glutathione (GSH) activity was assayed as described in [43]. Briefly, 100 µl sample (test), distilled water (Blank), and GSH (standard) were mixed with 100 µl of sulphosalicylic acid (4%), kept at 4 °C for at least 1 h, and then centrifuged at 1200 g for 10 min at 4 °C. 100 µl supernatant was then mixed with 2.7 ml phosphate buffer (0.1 M, pH 7.4) and 0.2 ml DTNB [5,5ʹ-dithiobis-(2-nitrobenzoic acid)] and incubated for 5 min. Measurements of the resulting yellow color were immediately conducted at 412 nm. A standard curve was constructed using standard GSH. Finally, GSH content was expressed as mg/mg protein.

The levels of superoxide dismutase (SOD) activity was evaluated as described in [44]. In the assay of SOD, 20 µl of sample (test) or buffer and 10 µl of pyrogallol (20 mM in 10 mM HCl) were added to 1 ml buffer solution. The sample absorbances for test (At) and reference (Ar) were measured against air after 30 min and 90 min at 420 nm. The percentage by which pyrogallol autoxidation was inhibited was calculated via: [% inhibition] = [100 – (At/min/ml sample)/(Ar/min/ml)] × 100. Using the SOD standard curve, one unit was found to equal 153 ng, so sample enzyme activity [U/mg protein] was calculated as specific activity divided by 153.

The levels of catalase (CAT) were assayed according to the method described in [45]. In the sample cuvette, 0.1 ml of sample was mixed with 0.5 ml of 0.2 M sodium phosphate buffer at pH 7.6 and 0.3 ml of 0.5% H_2_O_2_. The mixture was brought to a final volume of 3 ml with distilled water. The breakdown of H_2_O_2_ was recorded by measuring the absorbance at 240 nm and the enzyme activity was calculated as the change in absorbance per minute.

### Cytokine expression

Tumor necrosis factor-alpha (TNF-α) levels were estimated using ELISA-based cytokine kits of Global Headquarters according to [46]. Interleukin-6 (IL-6) was measured using MyBioSource ELISA kits as described in [47], and IL-10 was assayed using ThermoFisher ELISA kits according to [48].

### Histopathology

Fresh liver tissues from each group were fixed in 10% neutral formalin at room temperature for 24 h, dehydrated in ascending grades of ethanol, and embedded in paraffin. Tissue blocks were cut at 4 μm thick. Paraffin sections were stained with hematoxylin and eosin dye and examined using a digital light microscope (Olympus, Tokyo, Japan). The histological scoring was assessed according to López-Alonso [49]. A minimum of 3 slides for each animal and 10 fields per slide were examined and scored semi-quantitatively for the severity of changes. The scoring was done as none (−), mild (+), moderate (+ +), and severe (+ + +) changes.

### Statistical analysis

Data analysis was performed using SPSS v20.0 (Armonk, NY: IBM Corp). The Kolmogorov–Smirnov test was used to verify the normality of distribution. Quantitative data were described using range (minimum and maximum), mean, and standard deviation (SD). The used tests were *F* test (ANOVA) for normally distributed quantitative variables to compare between more than two groups, and Post Hoc Tukey test for pairwise comparisons. *p* values ≤ 0.05 were considered statistically significant.

## Results

### HPE regulates serum liver function

**Fig. 1:**
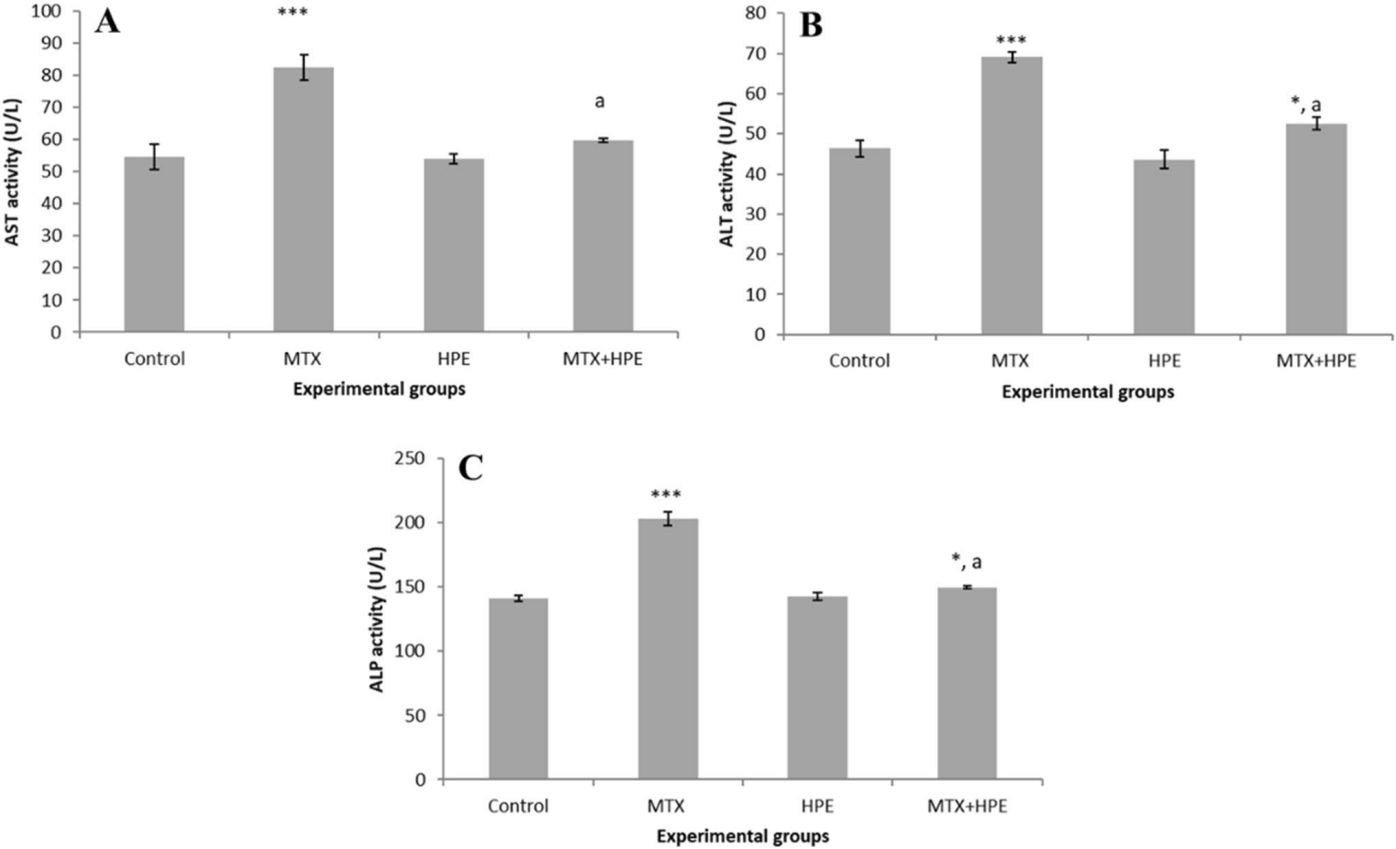
Effect of MTX and HPE administration on serum liver function for **A** AST, **B** ALT, and **C** ALP activity. Each value represents the mean ± SD for 10 animals per group. ***Highly significant in comparison with the control group at *p* < 0.001. *Significant in comparison with the control group at *p* < 0.05. ^a^Highly significant in comparison with the MTX group at *p* < 0.001

Data in Fig. 1 show the serum levels of AST, ALT, and ALP of all experimental groups. There were insignificant differences in the levels of AST, ALT, and ALP between the control group and HPE treated group. MTX-treated rats showed a highly significant increase (*p* < 0.001) in AST (82.5 ± 3.98), ALT (69.1 ± 1.38), and ALP (203.09 ± 5.43) levels compared with the control group. However, supplementation with both MTX and HPE resulted in a significant decrease in AST (59.75 ± 0.6), ALT (52.55 ± 1.64), and ALP (149.62 ± 1.05) levels relative to the MTX-treated group (*p* < 0.001), although a significant increase (*p* < 0.05) was still recorded in comparison with the control group.

### Lipid levels

**Fig. 2:**
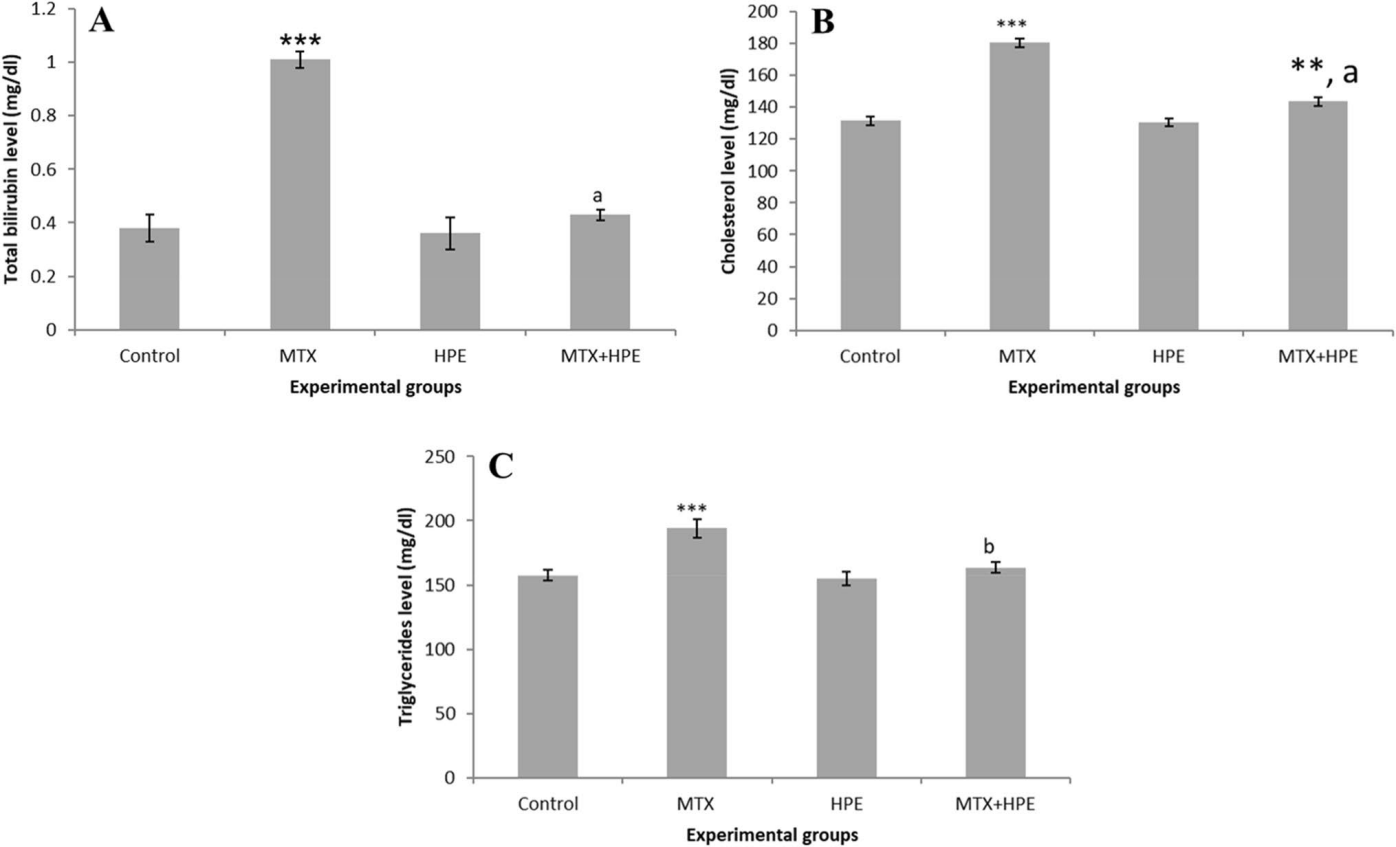
Effect of MTX and HPE administration on lipid levels of the different experimental groups for **A** total bilirubin, **B** cholesterol, and **C** triglycerides. Each value represents the mean ± SD for 10 animals per group. ***Highly significant in comparison with the control group at *p* < 0.001. **Significant in comparison with the control group at *p* < 0.01. ^a^Highly significant in comparison with the MTX group at *p* < 0.001. ^b^Significant in comparison with the MTX group at *p* < 0.01

Figure 2 shows the lipid profile levels of serum total bilirubin levels, total cholesterol, and triglycerides. Results exhibited insignificant differences between the control group and HPE treated group. When rats were treated with MTX, a highly significant rise (*p* < 0.001) in total bilirubin (1.01 ± 0.03), cholesterol (180.5 ± 2.59), and triglyceride (194 ± 6.92) levels were represented as compared with that of control group. On the other hand, we noted again that the combining effect of MTX and HPE resulted in highly significant (*p* < 0.001) reduction in the levels of total bilirubin (0.43 ± 0.02), cholesterol (143.5 ± 2.59), and triglycerides (163.5 ± 4.33) compared with the MTX group, although a significant increase (*p* < 0.01) was still recorded in comparison with the control group.

### HPE regulates lipid peroxidation and antioxidant activities

**Fig. 3:**
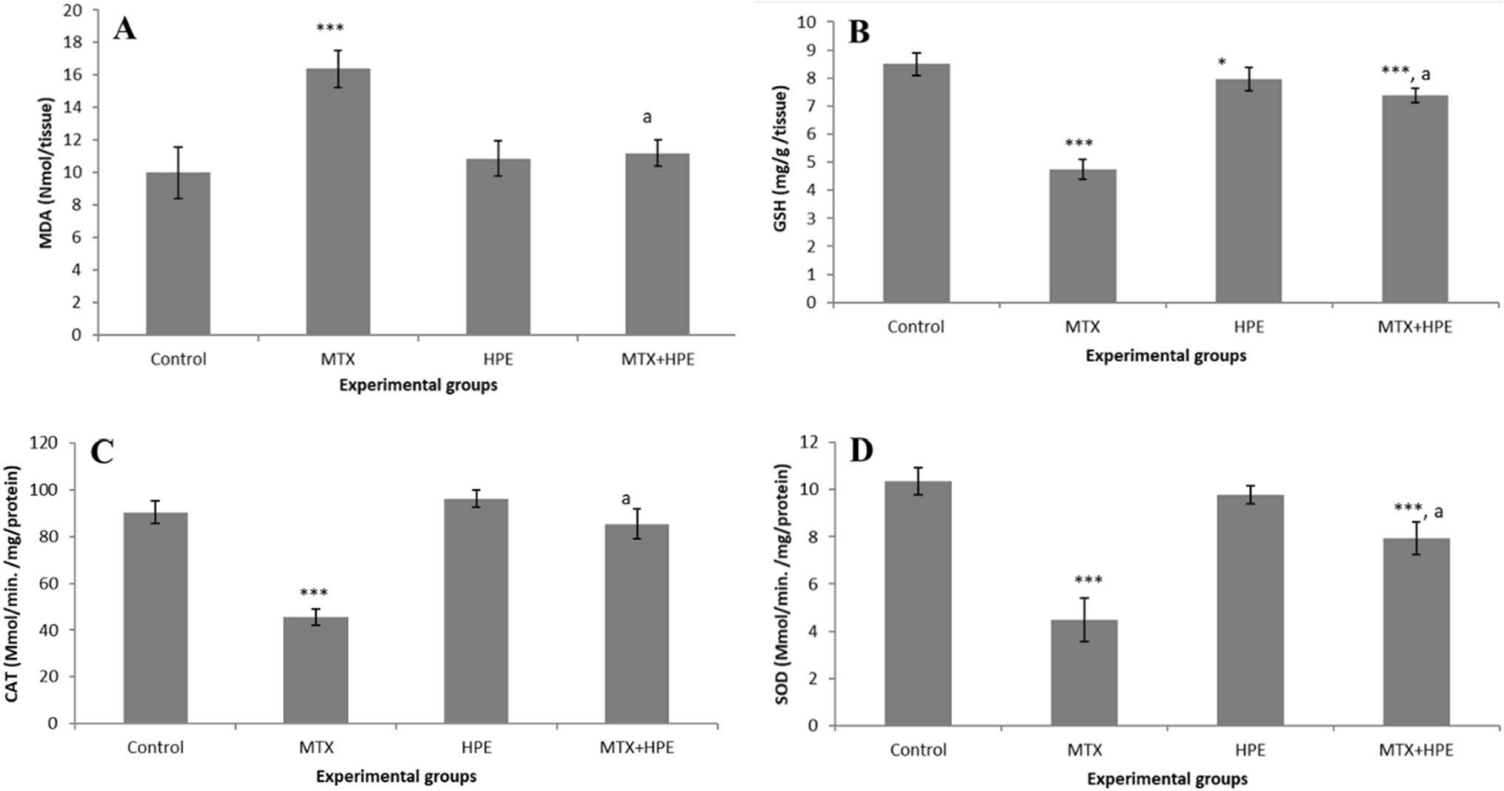
Effect of MTX and HPE administration on lipid peroxidation and antioxidant activities for **A** MDA, **B** GSH, **C** SOD, and **D** CAT. Each value represents the mean ± SD for 10 animals per group. ***Highly significant in comparison with the control group at *p* < 0.001. *Significant in comparison with the control group at *p* < 0.05. ^a^Highly significant in comparison with the MTX group at *p* < 0.001

The liver lipid peroxidation (MDA) and antioxidant markers (GSH, SOD, and CAT) are shown in Fig. 3. Data show that liver MDA concentration (16.4 ± 1.14) in MTX-treated rats was significantly higher (*p* < 0.001) as compared with control rats (Fig. 3A). However, supplementation with HPE counteracted the increase in MDA related to MTX. HPE significantly reduced the mean MDA values (11.2 ± 0.82) (*p* < 0.001). The effect of HPE administration on GSH levels was also examined in the liver of different groups. MTX-treated rats had a highly significant depletion (*p* < 0.001) in comparison with the control group (Fig. 3B), but HPE treatment significantly increased the GSH levels with value (7.38 ± 0.26). MTX administration significantly decreased the antioxidant enzyme activities of CAT (45.6 ± 0.35) (Fig. 3C) and SOD (4.48 ± 0.91) (Fig. 3D) in liver tissues in comparison with the control group, but data show a marked increase (*p* < 0.001) in tissue concentrations of CAT (85.4 ± 6.26) and SOD (7.94 ± 0.69) activity in liver tissues post exposure to HPE as compared with the MTX group.

### HPE regulates the expression of pro-inflammatory cytokines TNF-α, IL-6, and IL-10

**Fig. 4:**
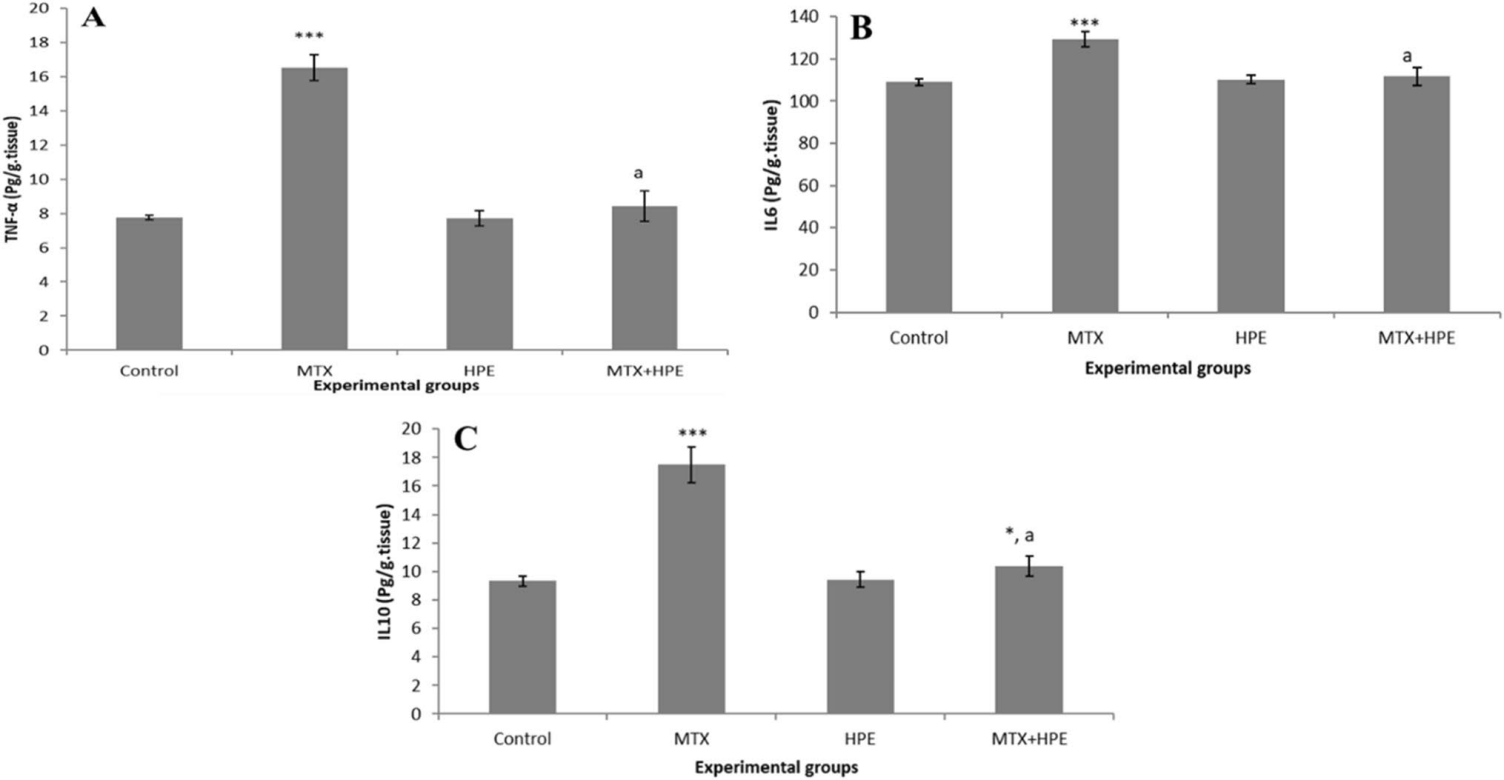
Effect of MTX and HPE administration on the expression of pro-inflammatory cytokines **A** TNF-α, **B** IL-6, and **C** IL-10. Each value represents the mean ± SD for 10 animals per group. ***Highly significant in comparison with the control group at *p* < 0.001. *Significant in comparison with the control group at *p* < 0.05. ^a^Highly significant in comparison with the MTX group at *p* < 0.001

Regarding pro- and anti-inflammatory markers, MTX injection caused significantly increased expression (*p* < 0.001) of TNF-α, IL-6, and IL-10 with values (16.54 ± 0.76, 129.4 ± 3.64 and 17.5 ± 1.25), respectively, in the liver tissues (Fig. 4). On the other hand, rats treated with HPE succeeded in counterbalancing this increase and returned to normal levels when compared to the MTX group.

### HPE protects hepatocytes against MTX‑induced hepatotoxicity

**Fig. 5:**
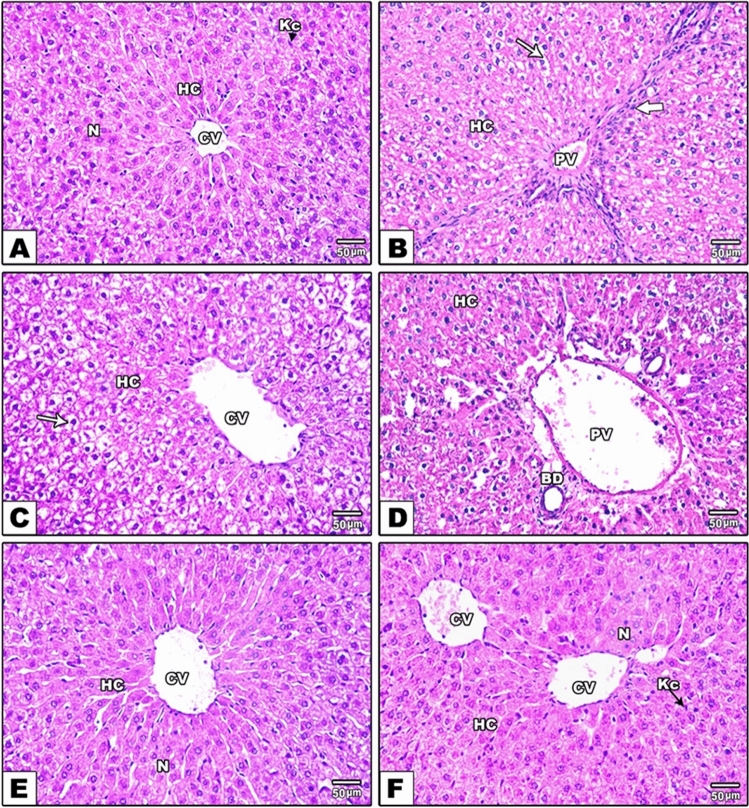
Effects of HPE treatment on histopathological injury in liver tissues following MTX-induced hepatotoxicity. **A** Control group showing normal histological architecture of the hepatocytes (HC); central vein (CV); and Kupffer cell (Kc). **B**, **C**, **D** MTX-treated group showing hepatocytes (HC) with cytoplasmic vacuolization (thin arrow); dilation for both central vein (CV) and portal vein (PV); inflammatory leucocytic infiltrations (thick arrow); and bile ducts (Bd) hyperplasia. **E** Rats treated with both MTX and HPE showed hepatocytes (HC) with normal nuclei (N) and central vein (CV). **F** HPE-treated rats showed normal hepatocytes (HC) with round basophilic nuclei (N) and normal Kupffer cell (Kc). Original Magnification: 200x

Hematoxylin and eosin (H&E) light micrographs of liver sections of rats are illustrated in Fig. 5. Photomicrographs from the control group show normal liver tissue architecture (Fig. 5A). The normal control structural unit of the liver is the hepatic lobule which appears roughly hexagonal in shape and is centered by a thin-walled vessel. Hepatocytes appear as large polygonal cells with round prominent nuclei. The space between these lobules contains capillaries and the liver sinusoids; these are irregularly dilated vessels in intimate contact with the hepatocytes. These sinusoids are lined by flat endothelial cells which could be readily distinguishable from hepatocytes by their flattened condensed nuclei and poorly stained cytoplasm. On the other hand, liver sections of MTX-treated rats revealed hepatocellular injury in some areas, as represented by the loss of the normal architecture of the liver tissues. Cytoplasmic vacuolation is evident. In addition, sinusoidal dilations and hyperemia (blood congestion) in sinusoids and central veins can be seen in Fig. 5B, as well as diffused and periportal leucocytic infiltration and increased numbers of Kupffer cells in Fig. 5C, D. The light microscopic examination of the liver tissues from rats treated with only HPE were found to be comparable to that of the control rats (Fig. 5E). However, screening of liver sections of rats treated with both MTX plus HPE showed minimal morphological changes in the histological features all over the liver tissue, with the hepatocytes appearing as typically polygonal with distinct cell margins and well-defined cytoplasm. The nuclei of the hepatocytes appeared to be normal. These results indicate an obvious protective role of HPE against the action of MTX (Fig. 5F). The histological scoring was graded and results are summarized in Table 1.

**Table 1:** Histopathological scoring analysis in liver sections of all experimental groups. Severity of liver histological changes using scores on a scale of none (–), mild ( +), moderate (+ +), and severe (+ + +) damage

Histological scoring	Control (G1)	MTX (G2)	HPE (G3)	MTX plus HPE (G4)
Irregular architecture	−	+ +	−	+
Portal inflammation	−	+ + +	−	−
Central vein dilation	−	+ + +	−	−
Sinusoidal dilation	−	+ +	−	−
Leukocytic infiltration	−	+ + +	−	−
Pyknotic nuclei	−	+ +	−	−
Cellular necrosis	−	+ +	−	−
Vacuolar degeneration	−	+	−	−
Portal vein dilation	−	+ + +	−	−

## Discussion

Methotrexate (MTX)-induced hepatotoxicity is a serious problem, because it affects MTX’s clinical therapeutic effects. Oxidative stress and lipid peroxidation mediated by oxygen free radicals have been considered as an important cause of MTX-induced neurotoxicity [8–11], hepatotoxicity [13, 14], intestinal toxicity [50], and nephrotoxicity [12]. The present study demonstrates the ability of human placenta extract (HPE) to ameliorate MTX-induced hepatotoxicity. MTX still remains one of the major causes of drug-induced steatohepatitis. Earlier studies investigated the cumulative dose of MTX-associated nonalcoholic fatty liver disease (NAFLD) with transaminitis, and it was found to have a significant positive correlation with the ALT level [23]. MTX reduces oxygen uptake and decreases oxidative phosphorylation in isolated mitochondria [51]; in addition, it inhibits several mitochondrial enzymes including 2-oxoglutarate, isocitrate, malate, and pyruvate dehydrogenases [52]. Treatment with HPE significantly reduced the biochemical and histological alterations induced by MTX in the liver of rats by regulating antioxidative responses and down-regulating MDA.

In the current study, MTX-treated rats demonstrated significantly increased levels of lipid peroxidation in the liver as exemplified by a significant increase in level of malondialdehyde (MDA) and a decrease in the antioxidants glutathione (GSH), catalase (CAT), and superoxide dismutase (SOD). These results are in accordance with others [25, 41, 42, 53–55]. It is of great interest to note that HPE supplementation resulted in a significant protective effect against MTX-induced lipid peroxidation. HPE reduced the level of MDA, reversed the reduction in GSH, and markedly increased the antioxidant enzyme activities of CAT and SOD in the livers of MTX-treated rats. Several studies on the anti-inflammatory and anti-oxidative activities of HPE have been conducted in different models, including porcine placenta extracts against contact dermatitis in vivo [56], benzo[a]pyrene-exposed rats [57] and concanavalin A-induced liver injury in mice [58]. In addition, other studies have shown that berberine, a natural isoquinoline alkaloid, can mitigate MTX-induced oxidative stress and inflammation in the liver tissue [59]. Excessive ROS generation by MTX can downregulate the antioxidant defense mechanism in the liver. Earlier studies showed exposure to MTX leads to upregulation of nuclear translocation of NF-κB and phosphorylated Iκ-B, down-regulation of antiapoptotic protein Bcl-2, and increased levels of caspase 3 that subsequently lead to cell death [60].

GSH is a protective endogenous antioxidant that plays an essential role in combatting free radicals and other oxidants. Our prior work [61] and that of others observed depletion of GSH levels in aged mice [62]. The current study shows that MTX-treated rats have significantly lower levels of antioxidant enzymes (GSH, CAT, and SOD) in comparison with the control group. Enzyme activation induced by HPE may increase the hydrogen peroxide to water conversion rate mediated by GPx and CAT as well as the superoxide to hydrogen peroxide conversion rate mediated by SOD [63, 64]. HPE treatment provided antioxidant effects not only on the non-enzymatic defense system (GSH) but also on the antioxidant enzyme activities of SOD and CAT, which have the capacity to neutralize free radicals and reduce cellular death and disease development. Thus, the use of HPE gives protection against various free radicals by inhibiting hydroxyl radicals and superoxide anions and by decreasing the lipid peroxidation level.

The correlation between MTX-related changes in lipid metabolism and oxidative stress has been of great interest. Earlier studies show how changes to the lipid profile result in increased oxidative stress. Increased lipid content is thought to result in decreased antioxidant enzyme expression, increased NADPH oxidase expression, and increased ROS concentrations [65]. Results in Figs. 1 and 2 show that MTX significantly altered the oxidant/antioxidant balance that caused severe liver injury, manifested as augmented activities of ALT, AST, ALP, serum total bilirubin levels, cholesterol, and triglycerides levels. These results are in accordance with others [66, 67]. HPE supplementation in MTX-treated rats resulted in a significant decrease in ALT, AST, and ALP activities relative to the MTX group. Furthermore, HPE-treated rats exhibited significant decreases in the concentrations of total bilirubin, total cholesterol, and triglycerides relative to the MTX group, suggesting that HPE may reduce oxidative stress by changing the lipid profile and indicating HPE’s ability to have a hepato-protective effect. In addition, the current study demonstrates that MTX induces pathological changes in the liver, including necrosis, inflammatory cell infiltration, hepatocyte apoptosis, degeneration, vacuolation, and bile duct hyperplasia. However, these hepatotoxic changes were significantly attenuated by HPE treatment, suggesting that HPE could effectively counteract MTX-induced liver cell injury.

The underlying mechanisms of HPE’s action is not clear, but it may involve its antioxidant and anti-inflammatory activities. HPE suppressed the expression of inflammation- and fibrosis-related genes and NADPH oxidase, especially in the perivascular regions. This suggests that reductions in oxidative stress and inflammation are key beneficial effects of HPE. These results are in accordance with [68]. Our observed results of HPE acting in concert with MTX matches closely with the recent study by Cataldi et al. of a new avenue for treatment and prevention of drug-induced steatosis and steatohepatitis [69], where they noted promising work in in vitro and animal studies of combining a hepatotoxic drug with a second molecule that aimed to antagonize liver toxicity as well as enhance the pharmacological activity of the drug. We do also note that while the present study reports on HPE’s antioxidant and anti-inflammatory activities, it is possible that HPE could potentially modify liver fat accumulation. This represents a main limitation of the current study and should be studied in the future.

In the current study, the MTX-treated group showed increased levels of TNF-α, IL-6, and IL-10 cytokines in the hepatic tissue, in agreement with others [53, 54]. This effect can be explained by [57, 67], who concluded that MTX-induced oxidative stress and lipid peroxidation caused degradation of IκB proteins and released NF-κB p65, the active subunit of NF-κB, in the cytoplasm. Translocation of NF-κB p65 to the nucleus promotes gene transcription of inflammatory cytokines. Our results showed that HPE supplementation decreased the production of TNF-α, IL-6, and IL-10, cytokines that play a central role in mediating inflammatory response. The imbalance of pro-inflammatory cytokines and anti-inflammatory cytokines may represent another important mechanism of liver injury.

The ability of HPE to protect hepatocytes against MTX‑induced hepatotoxicity is well illustrated by the H&E light micrographs of rats' liver sections shown in Fig. 5. Screening of liver sections of rats treated with both MTX plus HPE showed minimal morphological changes. The hepatocytes appeared as typically polygonal with distinct cell margins and well-defined cytoplasm, and the nuclei appeared to be normal. MTX produced severe cellular damage and inflammatory lesions in liver tissues, while treatment with HPE improved hepatic histologic architecture.

In conclusion, our results show that human placental extract (HPE) treatment ameliorates MTX-induced liver toxicity in rats. HPE exerts a hepatoprotective effect by improving the activity of endogenous antioxidant enzymes, increasing lipid peroxidation, and suppressing inflammatory responses.

## Data Availability

The data sets used and/or analyzed during the current study are available from the corresponding author on reasonable request.
